# Chorea: An unusual manifestation of endocrine diseases

**DOI:** 10.3389/fendo.2023.1155638

**Published:** 2023-03-03

**Authors:** Jia Zheng, Xiaohong Wu

**Affiliations:** Geriatric Medicine Center, Key Laboratory of Endocrine Gland Diseases of Zhejiang Province, Department of Endocrinology, Zhejiang Provincial People’s Hospital (Affiliated People’s Hospital, Hangzhou Medical College), Hangzhou, Zhejiang, China

**Keywords:** chorea, movement disorder, basal ganglia, endocrine, unusual manifestation

## Abstract

Chorea is a movement disorder involving involuntary movements of muscles of the face, neck, and limbs, usually caused by basal ganglia lesions. As an important part of the presentation of many neurological diseases, chorea is also an unusual manifestation of endocrine diseases and can be challenging to diagnose. Although the most common etiology of chorea is genetic, it is vital to identify acquired or symptomatic chorea, as these are potentially treatable conditions. This review summarizes the latest developments in various endocrine disease-related chorea, which will help clinicians to correctly identify and accurately treat it.

## 1 Introduction

**Figure 1:**
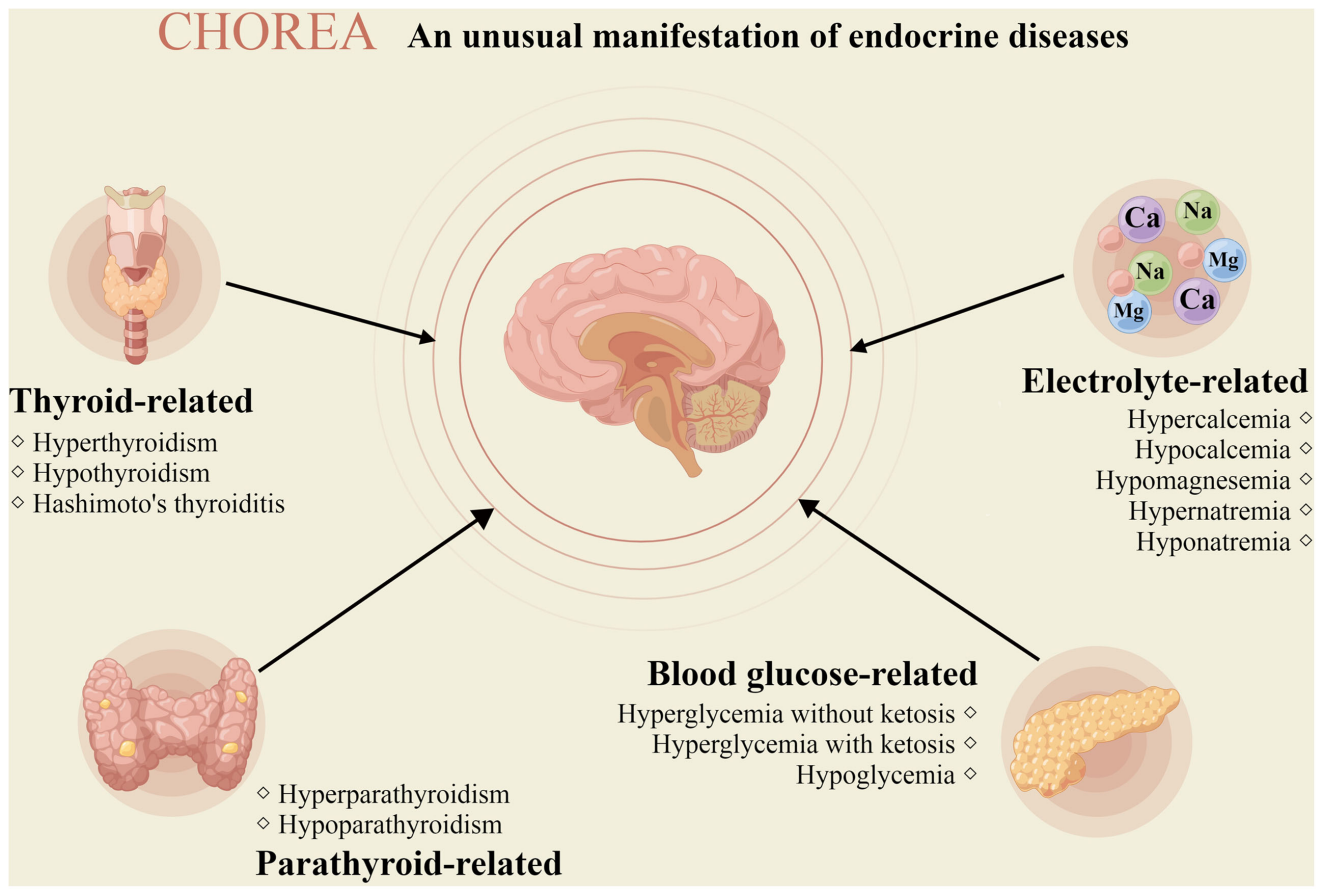
Chorea due to endocrine diseases. The picture depicts the different endocrine diseases that may manifest chorea (drawn by Figdraw).

Chorea refers to involuntary movements of limbs, trunk, neck, or face that rapidly flit from region to region in an irregular, flowing, non-stereotyped pattern (1). It is a relatively rare movement disorder characterized as involuntary, irregularly time, non-repetitive, purposeless, randomly distributed, and abrupt in character, which is commonly associated with Huntington’s disease or treatment with levodopa (2, 3). Therefore, we consider neurological diseases first when analyzing the etiology of chorea. However, chorea has recently been discovered to be a clinical manifestation of endocrine diseases such as hyperthyroidism and diabetes, and it may even be the initial symptom (4, 5). In clinical practice, endocrine disease-related chorea is uncommon and frequently misdiagnosed. The precise diagnosis of the etiology of chorea can better facilitate appropriate treatments and improve the life quality of patients (4, 6). This review summarizes the current research status of endocrine disease-related chorea (**Figure 1**), aiming to help clinicians correctly diagnose and precisely treat it.

## 2 Thyroid-related chorea

Thyroid metabolism plays an important role in human development, particularly the formation and functioning of the central and peripheral nervous systems. Thyroid diseases of genetic or acquired origin can lead to neurological disorders such as encephalopathy, myoclonus, and chorea (7, 8).

### 2.1 Hashimoto’s thyroiditis-related chorea

Dance-like movements caused by autoimmune encephalopathy associated with Hashimoto’s disease were first documented by Brain et al. (7) in 1966, and similar cases have occasionally been reported over the following two decades (9–12). Bilateral chorea is frequently one of the first symptoms in many patients, particularly adult females (13, 14). A prospective study investigated unexplained encephalopathy with detectable anti-thyroid antibodies, with an estimated prevalence of 2.1/100,000 (15). Chorea caused by Hashimoto’s encephalopathy is even rarer and difficult to evaluate (16).

The defining characteristics and pathogenesis of Hashimoto’s encephalopathy are still a common subject of debate. The mainstream view considers the condition a steroid-responsive encephalopathy associated with autoimmune thyroiditis and high titers of anti-thyroid antibodies, with or without thyroid dysfunction (9, 13, 17). The main hypotheses include the cerebral vasculitis theory (7, 18–21), the hormonal dysregulation theory (22–24), and the theory that autoantibodies act directly against various thyroid and extra-thyroid antigens.

High titers of anti-thyroid antibodies, particularly anti-thyroid peroxidase antibodies, are typically considered diagnostic. Although no characteristic neuroimaging signs are associated with the condition, other toxic, metabolic, and infectious causes of encephalopathy can be ruled out by neuroimaging. Most reported cases of Hashimoto’s thyroiditis-associated chorea have responded to a high-dose corticosteroid treatment, which is used as a criterion for disease definition (25–27). Treatment of Hashimoto’s thyroiditis-associated chorea usually consists of IV glucocorticoid for a few days followed by high-dose oral glucocorticoid tapered based on clinical improvement (28, 29). Most patients show a rapid response, but if left untreated or treatment is suspended, severe psychoneurological deficits may result.

### 2.2 Hyperthyroidism-related chorea

The most common neurological dysfunction associated with hyperthyroidism is tremor, but chorea, myoclonus, and spastic trunk flexion have also been reported (30–32). Chorea caused by hyperthyroidism or subclinical hyperthyroidism due to Graves’ disease, toxic multinodular goiter, or other medical and pharmacological origins is well-known but relatively rare, with a prevalence of less than 2% (33–35). The symptoms of chorea can be bilateral or unilateral (36–38). Its pathogenesis is unclear; the prevailing view is that hyperthyroidism-related chorea is related to complex dysfunction within the basal ganglia system. The mechanism could involve the alteration of dopamine metabolism in the striatum or alter basal ganglia function by affecting the metabolism of other neurotransmitters or inducing dysregulation of genes (35, 39, 40). No characteristic imaging signs have been associated with chorea caused by hyperthyroidism; instead, diagnosis typically involves the laboratory test results and imaging findings typical of hyperthyroidism combined with the effectiveness of the anti-hyperthyroid medication. In most patients, chorea symptoms gradually disappear when hyperthyroidism is controlled by using antithyroid drugs (34). At the same time, the use of drugs such as clonazepam and haloperidol can assist in the control of chorea symptoms (32, 35).

### 2.3 Hypothyroidism-related chorea

A close relationship exists between hypothyroidism and neurological deficits. Hereditary and acquired hypothyroidism can cause cognitive impairment, depression, and other neurological symptoms (41). After early screening and prophylactic treatment of congenital hypothyroidism, most children do not show any other signs. However, some children eventually develop choreoathetoid and respiratory diseases. This disorder is known as benign hereditary chorea (BHC) (42).

BHC is a rare autosomal dominant disorder caused by mutations in the NKX2-1 (TTF1 or TITF1) gene. More than 100 NKX2-1 mutations have been reported (43, 44). BHC usually develops in childhood, rarely in adolescence, and tends to resolve in adulthood (45). Approximately 30-50% of patients with NKX2-1 gene mutations have the classic brain-lung-thyroid triad (41), which manifests as neurological symptoms, such as chorea and dystonia in early infancy, along with abnormal thyroid function and respiratory diseases, such as neonatal respiratory distress syndrome and interstitial lung disease. This observation suggests that chorea and pulmonary symptoms in hypothyroid patients can be attributed to NKX2-1 gene mutations.

Domitille et al. (43) concluded that chorea in patients with BHC is mainly an isolated sign. Still, it may also be associated with dystonia, myoclonus, and tics, with chorea preceding hypotonia suggestive of BHC. Laboratory tests on patients with BHC may indicate abnormal thyroid function, including elevated levels of thyroid-stimulating hormone and reduced or normal levels of thyroxine. The neuroimaging findings of most patients with BHC are unremarkable. Abnormal pituitary saddle morphology was reported in seven patients with BHC (46). Although not all patients with BHC display consistent involvement of all three organs (brain, lung, and thyroid), many children develop interstitial lung disease. LeMoine et al. (47) suggested that high-resolution Computed Tomography of the thoracic region is helpful for disease diagnosis and that the most common imaging feature is ground-glass-like changes in the lungs. In conclusion, differential diagnosis of BHC remains challenging in many cases. Infantile chorea and its possible association with thyroid or lung disease may aid in differentiating BHC from other similar hereditary movement diseases, and patients should be proactively tested for NKX2-1 gene mutations.

Many treatment strategies have been developed for chorea, but most are largely ineffective, with only a few reporting effectiveness (48). Asmus et al. (49) reported that levodopa (20 mg/kg/d) significantly improved gait and reduced chorea in two patients. Nakamura et al. (50) reported that the dopamine agonist ropinirole hydrochloride (2 mg/d) reduced chorea in patients. Domitille et al. (43) reported beneficial effects of tetrabenazine in children (0.5 mg/kg/d) and adults (37.5 mg/d) on chorea and motor function. Gauquelin et al. (51) suggested that dyskinesia in BHC may respond to controlled-release methylphenidate hydrochloride (up to 30 mg/d). It is also recommended to use thyroxine replacement therapy for hypothyroidism and regular monitoring of thyroid function, and pulmonary symptoms should be treated symptomatically. Although no cure is available for BHC, supportive interventions can alleviate its manifestations.

## 3 Parathyroid-related chorea

Hyperparathyroidism-caused chorea was documented in one case (52); more information is missing, and the authenticity of the data is subject to verification. In contrast, hypoparathyroidism-caused chorea, including idiopathic hypoparathyroidism (53, 54), pseudohypoparathyroidism (55), and medically-induced hypoparathyroidism (56), is relatively common. Parathyroid dysfunction-related chorea is mainly caused by abnormalities in calcium, magnesium, and phosphorus metabolism, the mechanisms of which are described below. Hypoparathyroidism-caused chorea can be associated with the rare imaging finding of bilateral calcification in the basal ganglia (57). Hypothyroidism identification typically relies on a combination of relevant laboratory tests of parameters, such as parathyroid hormone and electrolyte levels, and questions about the patient’s medical history. After alleviating parathyroid dysfunction and impaired mineral metabolism by treated with vitamin D and calcium supplementation, chorea symptoms may be relieved (58).

## 4 Blood glucose-related chorea

Hyperglycemic non-ketotic hemichorea, known as diabetic striatopathy (DS), has gradually been recognized since Bidwell (59) first reported the development of hemiplegic chorea in patients with diabetes in 1960. Although the neurological manifestations of DS include stroke and peripheral neuropathy, chorea is relatively rare. Coupled with the fact that its imaging features can be easily misdiagnosed, its incidence is likely underestimated at approximately <1/100,000 (60). The disease is common in older Asian women with type 2 diabetes (61, 62). A recent meta-analysis suggested that 96.6% of 176 patients with DS had type 2 diabetes. Patients from Asia accounted for 71.6% of the total sample, followed by Europe (8.5%) and America (4%). The average age was 67.6 ± 15.9 years, with a male-to-female ratio of 1:1.7 (63).

The pathogenesis of DS is currently believed to involve ischemic hemorrhage (64, 65), metabolic disturbances (66, 67), ionic deposition (62, 68, 69), autoimmune inflammatory responses (70–72), neurodegeneration (73, 74), dopaminergic and estrogenic alterations (75, 76), and genetic susceptibility (77). Patients with DS tend to present with unilateral symptoms. In addition, lateralized chorea can occur in patients with hyperglycemic ketosis (78, 79) or hypoglycemia (80); thus, it cannot be attributed to a single cause.

Imaging often shows high density on Computed Tomography scans of the striatum and high signal intensity on Magnetic Resonance Imaging (T1-weighted image) (81–83). However, patients may present with chorea symptoms in the absence of imaging abnormalities or vice versa. Therefore, imaging studies must be performed with a larger sample size and long-term follow-up. Diagnosis of DS is typically one of exclusion; other causes can be excluded in patients with elevated blood glucose and glycated hemoglobin levels, negative urinary ketone bodies, and no differences in the levels of electrolytes, autoantibodies, rheumatoid immune markers, thyroid function, serum copper, and copper cyanide.

DS Treatment primarily involves blood glucose control, and most patients experience gradual improvement over several days or weeks. If chorea symptoms do not resolve in the absence of hyperglycemia, haloperidol, pimozide, and other dopamine receptor antagonists can be administered (63, 78, 81, 84). However, as dopamine receptor antagonists can cause delayed dyskinesia, treatment dosage should be tailored to meet individual requirements.

## 5 Electrolyte-related chorea

Changes in electrolyte levels may affect brain areas with high metabolic rates, such as the basal ganglia, which can trigger a range of neurological symptoms. However, these changes are reversible in the absence of structural damage (5).

### 5.1 Hypercalcemia or hypocalcemia

Hypercalcemia causes chorea by stimulating dopamine release, although this is relatively rare (85). Severe hypocalcemia can increase excitability in the brain, which may result in neurological symptoms such as tics, seizures, delirium, and, in rare cases, chorea (86). Hypocalcemia-induced chorea is most commonly observed in hypoparathyroidism (87). Other reported causes of chorea secondary to hypocalcemia include malabsorption and bisphosphonate therapy (88–90). The mechanism by which hypocalcemia causes extrapyramidal symptoms remains unknown. Calcium deposits in the basal ganglia were initially believed to be responsible for chorea; however, it is usually systemic, and patients may exhibit other manifestations of hypocalcemia. Chorea typically resolves once blood calcium levels are normalized, but dopamine modulators may occasionally be required (91).

### 5.2 Hypomagnesemia

Hypomagnesemia may cause neurological symptoms similar to hypocalcemia, but chorea is uncommon and usually occurs in the context of other neurological signs (4, 92). The main causes of hypomagnesemia are inadequate oral intake, diarrhea, kidney disease, diuresis, acute pancreatitis, and hypercalcemia. Its treatment focuses on the correction of magnesium deficiency. On the one hand, the prevention and treatment of the original disease, in order to remove the cause of low magnesium. On the other hand, supplement magnesium, mostly magnesium sulfate preparations are used.

### 5.3 Hypernatremia or hyponatremia

Sodium excess-induced hypernatremia may cause neurological symptoms ranging from impaired consciousness to dystonia or chorea (91, 93). Although hypernatremia is a common electrolyte disorder in medical practice, hypernatremia-caused chorea is extremely rare; only isolated cases have been reported, and adults and children can develop the disease (93). The underlying mechanism may involve the dehydration of nerve cells due to osmotic imbalance or lysis of myelin sheaths outside the pons (94, 95). Treatment is often given with 5 percent glucose and water (93). With the correction of hypernatremia, chorea symptoms gradually decrease.

Chorea may also occur during the hyponatremic phase or after alleviating electrolyte disturbances. Chorea associated with hyponatremia has been reported in intracranial tuberculomas. Rapid alleviation of hyponatremia can cause the lysis of the central and extra-pontine myelin sheaths, which may also result in motor deficits (91, 96–99). In all the metabolic cases, the chorea resolved after the blood parameters normalized (93).

## 6 The possible common mechanisms

### 6.1 Autoimmune response

Autoimmune chorea is one of the major causes of adult chorea. It is associated with various antibodies, including thyroid peroxidase, thyroglobulin or thyroid microsomal thyroid autoantibodies, glutamic acid decarboxylase 65-kDa isoform antibodies, striatal antibodies, and voltage-gated calcium channel antibodies (41, 100). Autoimmune thyroiditis-associated steroid-responsive encephalopathy is associated with high titers of antithyroid antibodies. Glutamic acid decarboxylase 65-kDa isoform antibodies, usually at low titers, are also associated with other autoimmune disorders, such as type 1 diabetes, pernicious anemia, and autoimmune thyroiditis (101). Ghosh et al. (102) reported a case of diabetes mellitus with COVID-19 and chorea. They suggested that SARS-COV-2 infection could induce diabetic ketoacidosis and damage the striatum by infecting cell metabolism and inducing immune cell aggregation. These disorders are mediated by abnormal immune attacks that lead to neuronal dysfunction and manifest as chorea.

### 6.2 Hypersensitivity of the dopaminergic system

The dopamine system in the nigrostriatal pathway of the basal ganglia is closely related to chorea. Hypersensitivity of the dopaminergic system is one of the potential mechanisms of hyperthyroidism-associated chorea. Homo-vanillic acid, a dopamine metabolite, was significantly decreased in the cerebrospinal fluid of hyperthyroid patients (103). Moreover, treatment with dopamine antagonists can alleviate the symptoms of hyperthyroidism-related chorea (104). Hyperglycemia induces increased sensitivity of striatal dopamine receptors, and prolonged hyperglycemia leads to dysregulation of vascular autoregulation and interferes with basal ganglia dopamine metabolism, leading to chorea (78, 105).

### 6.3 Alteration of the blood-brain barrier

Multiple factors contribute to altered blood-brain barrier permeability, exacerbating basal ganglia injury. Calcium acts as a second messenger, and sustained dysregulation of cellular calcium homeostasis leads to the collapse of cellular function and structure (106). Altered calcium homeostasis usually precedes striatal dysfunction. In addition, calcium dysregulation can cause morphological and functional changes in neurons (107). And sodium, as an important electrolyte for regulating osmotic pressure in the brain, can cause a series of neurological symptoms, such as chorea, due to blood-brain barrier alteration, mainly in the case of rapid correction of hyponatremia. In hyponatremia, brain tissue is in a hypotonic state, and rapid supplementation of hypertonic saline can cause a rapid increase in plasma osmolality, resulting in brain tissue dehydration and blood-brain barrier disruption (93, 108). Hyperglycemia also exacerbates basal ganglia injury by increasing blood-brain barrier permeability (105).

## 7 Conclusion

When meeting with patients with chorea, clinicians should direct detailed laboratory tests, imaging, comprehensive neurological examination, and recording patients’ medical history, aiming to guide the comprehensive diagnosis of chorea etiology (shown in **Table 1**). Although endocrine disease-induced chorea is less common than other neurological disorders, recognizing the clinical features of these conditions can reduce misdiagnosis and aid in early diagnosis and treatment of disease. What are the reasons for the differences in the manifestations of chorea caused by different endocrine diseases? Whether there are specific biomarkers and imaging features for endocrine disease-related chorea? These unresolved issues need further research.

**Table 1:** What to measure when clinicians first meeting patients with chorea.

Examination items	Specific checklist		
History-taking	• Chief complaints • Current medical history • Past medical history • Personal history • Family history		
Neurological examination	• General physical examination • State of consciousness examination • Mental state examination • Cranial nerves examination • Motion system examination	• Sensory system examination • Reflexes examination • Signs of meningeal irritation examination • Autonomic examination	
Laboratory tests	• Blood • Urine • Serum sodium • Serum potassium • Serum calcium • Serum magnesium • Serum creatinine • Blood urea nitrogen	• Urine albumin-to-creatinine ratio • Blood osmolality concentration • Liver function • Serum lipids • Fasting glucose • Postprandial glucose • Glycated hemoglobin • Thyroid function and antibodies	• Human immunodeficiency virus • Syphilis • Hepatitis • Copper blue protein • Antiphospholipid antibodies • Anti-streptococcal hemolysin O antibodies • Tumor markers
Imaging	• Cranial CT • Cranial MRI • Electroencephalogram		

## Author contributions

JZ wrote the original draft. XW reviewed, edited and supervised the entire paper. All authors have read and agreed to the published version of the manuscript. All authors contributed to the article and approved the submitted version.

## References

[1] Walker RH. Differential diagnosis of chorea. Curr Neurol Neurosci Rep (2011) 11(4):385–95. doi: 10.1007/s11910-011-0202-2 21465146

[2] Bovenzi R Conti M Cerroni R Pierantozzi M Stefani A Pisani A. Adult-onset sporadic chorea: Real-world data from a single-centre retrospective study. Neurol Sci (2022) 43(1):387–92. doi: 10.1007/s10072-021-05332-w PMC8724109 34041635

[3] Cossu G Colosimo C. Hyperkinetic movement disorder emergencies. Curr Neurol Neurosci Rep (2017) 17(1):6. doi: 10.1007/s11910-017-0712-7 28168537

[4] Hermann A Walker RH. Diagnosis and treatment of chorea syndromes. Curr Neurol Neurosci Rep (2015) 15(2):514. doi: 10.1007/s11910-014-0514-0 25620691

[5] Janavs JL Aminoff MJ. Dystonia and chorea in acquired systemic disorders. J Neurol Neurosurg Psychiatry (1998) 65(4):436–45. doi: 10.1136/jnnp.65.4.436 PMC2170280 9771763

[6] Walker RH. The non-huntington disease choreas: Five new things. Neurol Clin Pract (2016) 6(2):150–6. doi: 10.1212/CPJ.0000000000000236 PMC5720620 29377035

[7] Brain L Jellinek EH Ball K. Hashimoto's disease and encephalopathy. Lancet (1966) 2(7462):512–4. doi: 10.1016/s0140-6736(66)92876-5 4161638

[8] Park J Kim JG Park SP Lee HW. Asymmetric chorea as presenting symptom in graves' disease. Neurol Sci (2012) 33(2):343–5. doi: 10.1007/s10072-011-0679-0 21796431

[9] Liu MY Zhang SQ Hao Y Zheng HM. Paroxysmal kinesigenic dyskinesia as the initial symptom of hashimoto encephalopathy. CNS Neurosci Ther (2012) 18(3):271–3. doi: 10.1111/j.1755-5949.2012.00297.x PMC6493638 22449112

[10] Sharan A Sengupta S Mukhopadhyay S Ghosh B. Hashimoto's encephalopathy presenting with chorea. J Assoc Physicians India (2015) 63(9):83–4. doi: 10.4103/0028-3886.170064 27608878

[11] Taurin G Golfier V Pinel JF Deburghgraeve V Poirier JY Edan G. Choreic syndrome due to hashimoto's encephalopathy. Mov Disord (2002) 17(5):1091–2. doi: 10.1002/mds.10230 12360567

[12] Yu H Qiu H Pan J Wang S Bao Y Jia W. Hashimoto's thyroiditis concomitant with sequential autoimmune hepatitis, chorea and polyserositis: A new entity of autoimmune polyendocrine syndrome? Intern Med (2013) 52(2):255–8. doi: 10.2169/internalmedicine.52.6799 23318858

[13] Chaudhuri A Behan PO. The clinical spectrum, diagnosis, pathogenesis and treatment of hashimoto's encephalopathy (recurrent acute disseminated encephalomyelitis). Curr Med Chem (2003) 10(19):1945–53. doi: 10.2174/0929867033456945 12871097

[14] Galluzzi S Geroldi C Zanetti O Frisoni GB. Hashimoto's encephalopathy in the elderly: Relationship to cognitive impairment. J Geriatr Psychiatry Neurol (2002) 15(3):175–9. doi: 10.1177/089198870201500309 12230088

[15] Ferracci F Bertiato G Moretto G. Hashimoto's encephalopathy: Epidemiologic data and pathogenetic considerations. J Neurol Sci (2004) 217(2):165–8. doi: 10.1016/j.jns.2003.09.007 14706219

[16] Churilov LP Sobolevskaia PA Stroev YI. Thyroid gland and brain: Enigma of hashimoto's encephalopathy. Best Pract Res Clin Endocrinol Metab (2019) 33(6):101364. doi: 10.1016/j.beem.2019.101364 31801687

[17] Chong JY Rowland LP Utiger RD. Hashimoto encephalopathy: syndrome or myth? Arch Neurol (2003) 60(2):164–71. doi: 10.1001/archneur.60.2.164 12580699

[18] Sue CM Fung V Halpern JP Boyages SC Yiannikas C. Hashimoto's encephalopathy. J Clin Neurosci (1997) 4(1):74–7. doi: 10.1016/s0967-5868(97)90018-7 18638931

[19] Jellinek EH Ball K. Letter: Hashimoto's disease, encephalopathy, and splenic atrophy. Lancet (1976) 1(7971):1248. doi: 10.1016/s0140-6736(76)92207-8 58299

[20] Ferracci F Moretto G Candeago RM Cimini N Conte F Gentile M. Antithyroid antibodies in the CSF: their role in the pathogenesis of hashimoto's encephalopathy. Neurology (2003) 60(4):712–4. doi: 10.1212/01.wnl.0000048660.71390.c6 12601119

[21] Bertoni M Falcini M Sestini S Niccoli L Nannini C Cantini F. Encephalopathy associated with hashimoto's thyroiditis: an additional case. Eur J Intern Med (2003) 14(7):434–7. doi: 10.1016/j.ejim.2003.06.002 14614977

[22] Ghawche F Bordet R Destee A. Hashimoto's encephalopathy: toxic or autoimmune mechanism? Rev Neurol (Paris) (1992) 148(5):371–3. 1448653

[23] Latinville D Bernardi O Cougoule JP Bioulac B Henry P Loiseau P. Hashimoto's thyroiditis and myoclonic encephalopathy. Pathogenic hypothesis. Rev Neurol (Paris) (1985) 141(1):55–8. 3920744

[24] Maeda K Tanimoto K. Epileptic seizures induced by thyrotropin releasing hormone. Lancet (1981) 1(8228):1058–9. doi: 10.1016/s0140-6736(81)92226-1 6112439

[25] Castillo P Woodruff B Caselli R Vernino S Lucchinetti C Swanson J. Steroid-responsive encephalopathy associated with autoimmune thyroiditis. Arch Neurol (2006) 63(2):197–202. doi: 10.1001/archneur.63.2.197 16476807

[26] Fatourechi V. Hashimoto's encephalopathy: myth or reality? an endocrinologist's perspective. Best Pract Res Clin Endocrinol Metab (2005) 19(1):53–66. doi: 10.1016/j.beem.2004.11.006 15826922

[27] Jacob S Rajabally YA. Hashimoto's encephalopathy: steroid resistance and response to intravenous immunoglobulins. J Neurol Neurosurg Psychiatry (2005) 76(3):455–6. doi: 10.1136/jnnp.2004.049395 PMC1739535 15716552

[28] Gauthier AC Baehring JM. Hashimoto's encephalopathy mimicking Creutzfeldt-Jakob disease. J Clin Neurosci (2017) 35:72–3. doi: 10.1016/j.jocn.2016.09.019 27743761

[29] Patnaik SK Upreti V Dhull P. Steroid responsive encephalopathy associated with autoimmune thyroiditis (SREAT) in childhood. J Pediatr Endocrinol Metab (2014) 27(7-8):737–44. doi: 10.1515/jpem-2013-0435 24598831

[30] Loh LM Hum AY Teoh HL Lim EC. Graves' disease associated with spasmodic truncal flexion. Parkinsonism Relat Disord (2005) 11(2):117–9. doi: 10.1016/j.parkreldis.2004.08.004 15734671

[31] Ross DS Burch HB Cooper DS Greenlee MC Laurberg P Maia AL. 2016 American Thyroid association guidelines for diagnosis and management of hyperthyroidism and other causes of thyrotoxicosis. Thyroid (2016) 26(10):1343–421. doi: 10.1089/thy.2016.0229 27521067

[32] Teoh HL Lim EC. Platysmal myoclonus in subclinical hyperthyroidism. Mov Disord (2005) 20(8):1064–5. doi: 10.1002/mds.20524 15929096

[33] Kondziella D Brederlau A Asztely F. Choreathetosis due to abuse of levothyroxine. J Neurol (2009) 256(12):2106–8. doi: 10.1007/s00415-009-5314-0 19763382

[34] Masannat Y Gandhy R Olajide O Kheetan R Yaqub A. Chorea associated with thyrotoxicosis due to toxic multinodular goiter. Thyroid (2011) 21(11):1279–80. doi: 10.1089/thy.2011.0018 21936672

[35] Yu JH Weng YM. Acute chorea as a presentation of graves disease: Case report and review. Am J Emerg Med (2009) 27(3):369 e1– e3. doi: 10.1016/j.ajem.2008.05.031 19328390

[36] Lun R Moores M Mestre T Breiner A. Thyrotoxicosis resulting in unilateral upper limb chorea and ballismus. Can J Neurol Sci (2022) 49(3):431–2. doi: 10.1017/cjn.2021.136 34134800

[37] Miao J Liu R Li J Du Y Zhang W Li Z. Meige's syndrome and hemichorea associated with hyperthyroidism. J Neurol Sci (2010) 288(1-2):175–7. doi: 10.1016/j.jns.2009.10.018 19883923

[38] Muthipeedika JMA Moosa A Kumar A Suchowersky O. Bilateral chorea–ballism associated with hyperthyroidism. Mov Disord (2005) 20(4):512. doi: 10.1002/mds.20436 15732125

[39] Garcin B Louissaint T Hosseini H Blanc R Fenelon G. Reversible chorea in association with graves' disease and moyamoya syndrome. Mov Disord (2008) 23(4):620–2. doi: 10.1002/mds.21941 18186119

[40] Leblicq C Duval M Carmant L Van Vliet G Alos N. Rising serum thyroxine levels and chorea in graves' disease. Pediatrics (2013) 131(2):e616–9. doi: 10.1542/peds.2012-0686 23296438

[41] Kurian MA Jungbluth H. Genetic disorders of thyroid metabolism and brain development. Dev Med Child Neurol (2014) 56(7):627–34. doi: 10.1111/dmcn.12445 PMC4231219 24665922

[42] Inzelberg R Weinberger M Gak E. Benign hereditary chorea: an update. Parkinsonism Relat Disord (2011) 17(5):301–7. doi: 10.1016/j.parkreldis.2011.01.002 21292530

[43] Gras D Jonard L Roze E Chantot-Bastaraud S Koht J Motte J. Benign hereditary chorea: phenotype, prognosis, therapeutic outcome and long term follow-up in a large series with new mutations in the TITF1/NKX2-1 gene. J Neurol Neurosurg Psychiatry (2012) 83(10):956–62. doi: 10.1136/jnnp-2012-302505 22832740

[44] Moya CM Zaballos MA Garzon L Luna C Simon R Yaffe MB. TAZ/WWTR1 mediates the pulmonary effects of NKX2-1 mutations in brain-Lung-Thyroid syndrome. J Clin Endocrinol Metab (2018) 103(3):839–52. doi: 10.1210/jc.2017-01241 29294041

[45] Carre A Szinnai G Castanet M Sura-Trueba S Tron E Broutin-L'Hermite I. Five new TTF1/NKX2.1 mutations in brain-lung-thyroid syndrome: rescue by PAX8 synergism in one case. Hum Mol Genet (2009) 18(12):2266–76. doi: 10.1093/hmg/ddp162 19336474

[46] Thust S Veneziano L Parkinson MH Bhatia KP Mantuano E Gonzalez-Robles C. Altered pituitary morphology as a sign of benign hereditary chorea caused by TITF1/NKX2.1 mutations. Neurogenetics (2022) 23(2):91–102. doi: 10.1007/s10048-021-00680-3 35079915 PMC8960566

[47] LeMoine BD Browne LP Liptzin DR Deterding RR Galambos C Weinman JP. High-resolution computed tomography findings of thyroid transcription factor 1 deficiency (NKX2-1 mutations). Pediatr Radiol (2019) 49(7):869–75. doi: 10.1007/s00247-019-04388-3 30927038

[48] Parnes M Bashir H Jankovic J. Is benign hereditary chorea really benign? brain-Lung-Thyroid syndrome caused by NKX2-1 mutations. Mov Disord Clin Pract (2019) 6(1):34–9. doi: 10.1002/mdc3.12690 PMC6335533 30746413

[49] Asmus F Horber V Pohlenz J Schwabe D Zimprich A Munz M. A novel TITF-1 mutation causes benign hereditary chorea with response to levodopa. Neurology (2005) 64(11):1952–4. doi: 10.1212/01.WNL.0000164000.75046.CC 15955952

[50] Nakamura K Sekijima Y Nagamatsu K Yoshida K Ikeda S. A novel nonsense mutation in the TITF-1 gene in a Japanese family with benign hereditary chorea. J Neurol Sci (2012) 313(1-2):189–92. doi: 10.1016/j.jns.2011.09.013 21982616

[51] Gauquelin L Tran LT Chouinard S Bernard G. The movement disorder of brain-Lung-Thyroid syndrome can be responsive to methylphenidate. Tremor Other Hyperkinet Mov (N Y) (2017) 7:508. doi: 10.7916/D84X5M9Z 29109906 PMC5666014

[52] Rizzo GN Olanow CW Roses AD. Chorea in hyperparathyroidism. report of a case. AMB Rev Assoc Med Bras (1981) 27(5):155–6. 6976597

[53] Hossain M. Neurological and psychiatric manifestations in idiopathic hypoparathyroidism: Response to treatment. J Neurol Neurosurg Psychiatry (1970) 33(2):153–6. doi: 10.1136/jnnp.33.2.153 PMC493434 5443473

[54] Kato H Kobayashi K Kohari S Okita N Iijima K. Paroxysmal kinesigenic choreoathetosis and paroxysmal dystonic choreoathetosis in a patient with familial idiopathic hypoparathyroidism. Tohoku J Exp Med (1987) 151(2):233–9. doi: 10.1620/tjem.151.233 3576617

[55] Thomas KP Muthugovindan D Singer HS. Paroxysmal kinesigenic dyskinesias and pseudohypo-parathyroidism type ib. Pediatr Neurol (2010) 43(1):61–4. doi: 10.1016/j.pediatrneurol.2010.03.012 20682207

[56] Salti I Faris A Tannir N Khouri K. Rapid correction by 1-alpha-hydroxycholecalciferol of hemichorea in surgical hypoparathyroidism. J Neurol Neurosurg Psychiatry (1982) 45(1):89–90. doi: 10.1136/jnnp.45.1.89 7062078 PMC491274

[57] Saleem S Aslam HM Anwar M Anwar S Saleem M Saleem A. Fahr's syndrome: Literature review of current evidence. Orphanet J Rare Dis (2013) 8:156. doi: 10.1186/1750-1172-8-156 24098952 PMC3853434

[58] Galvez-Jimenez N Hanson MR Cabral J. Dopa-resistant parkinsonism, oculomotor disturbances, chorea, mirror movements, dyspraxia, and dementia: the expanding clinical spectrum of hypoparathyroidism. A Case Rep Mov Disord (2000) 15(6):1273–6. doi: 10.1002/1531-8257(200011)15:6<1273::aid-mds1038>3.0.co;2-o 11104223

[59] Bedwell SF. Some observations on hemiballismus. Neurology (1960) 10:619–22. doi: 10.1212/wnl.10.6.619 13798206

[60] Ondo WG. Hyperglycemic nonketotic states and other metabolic imbalances. Handb Clin Neurol (2011) 100:287–91. doi: 10.1016/B978-0-444-52014-2.00021-5 21496588

[61] Cosentino C Torres L Nunez Y Suarez R Velez M Flores M. Hemichorea/Hemiballism associated with hyperglycemia: Report of 20 cases. Tremor Other Hyperkinet Mov (N Y) (2016) 6:402. doi: 10.7916/D8DN454P 27536463 PMC4955070

[62] Oh SH Lee KY Im JH Lee MS. Chorea associated with non-ketotic hyperglycemia and hyperintensity basal ganglia lesion on T1-weighted brain MRI study: a meta-analysis of 53 cases including four present cases. J Neurol Sci (2002) 200(1-2):57–62. doi: 10.1016/s0022-510x(02)00133-8 12127677

[63] Chua CB Sun CK Hsu CW Tai YC Liang CY Tsai IT. "Diabetic striatopathy": clinical presentations, controversy, pathogenesis, treatments, and outcomes. Sci Rep (2020) 10(1):1594. doi: 10.1038/s41598-020-58555-w 32005905 PMC6994507

[64] Chang KH Tsou JC Chen ST Ro LS Lyu RK Chang HS. Temporal features of magnetic resonance imaging and spectroscopy in non-ketotic hyperglycemic chorea-ballism patients. Eur J Neurol (2010) 17(4):589–93. doi: 10.1111/j.1468-1331.2009.02867.x 20039938

[65] Zaitout ZCT. And MRI findings in the basal ganglia in non-ketotic hyperglycaemia associated hemichorea and hemi-ballismus (HC-HB). Neuroradiology (2012) 54(10):1119–20. doi: 10.1007/s00234-012-1021-0 22383069

[66] Aquino JH Spitz M Pereira JS. Hemichorea-hemiballismus as the first sign of type 1b diabetes during adolescence and its recurrence in the setting of infection. J Child Neurol (2015) 30(10):1362–5. doi: 10.1177/0883073814553972 25387546

[67] Chang X Hong W Yu H Yao Y. Chorea associated with nonketotic hyperglycemia: A case report with atypical imaging changes. Med (Baltimore) (2017) 96(45):e8602. doi: 10.1097/MD.0000000000008602 PMC5690779 29137086

[68] Cherian A Thomas B Baheti NN Chemmanam T Kesavadas C. Concepts and controversies in nonketotic hyperglycemia-induced hemichorea: further evidence from susceptibility-weighted MR imaging. J Magn Reson Imaging (2009) 29(3):699–703. doi: 10.1002/jmri.21672 19243044

[69] Mitchell K Kariko K Harris VA Rangel Y Keller JM Welsh FA. Preconditioning with cortical spreading depression does not upregulate Cu/Zn-SOD or Mn-SOD in the cerebral cortex of rats. Brain Res Mol Brain Res (2001) 96(1-2):50–8. doi: 10.1016/s0169-328x(01)00266-2 11731008

[70] Ahlskog JE Nishino H Evidente VG Tulloch JW Forbes GS Caviness JN. Persistent chorea triggered by hyperglycemic crisis in diabetics. Mov Disord (2001) 16(5):890–8. doi: 10.1002/mds.1171 11746619

[71] Chang CV Felicio AC Godeiro Cde O Jr. Matsubara LS Duarte DR Ferraz HB. Chorea-ballism as a manifestation of decompensated type 2 diabetes mellitus. Am J Med Sci (2007) 333(3):175–7. doi: 10.1097/MAJ.0b013e3180318e34 17496737

[72] Wang JH Wu T Deng BQ Zhang YW Zhang P Wang ZK. Hemichorea-hemiballismus associated with nonketotic hyperglycemia: a possible role of inflammation. J Neurol Sci (2009) 284(1-2):198–202. doi: 10.1016/j.jns.2009.04.005 19428031

[73] Sharma R Buras E Terashima T Serrano F Massaad CA Hu L. Hyperglycemia induces oxidative stress and impairs axonal transport rates in mice. PLoS One (2010) 5(10):e13463. doi: 10.1371/journal.pone.0013463 20976160 PMC2956689

[74] Zheng W Chen L Chen JH Lin X Tang Y Lin XJ. Hemichorea associated with non-ketotic hyperglycemia: A case report and literature review. Front Neurol (2020) 11:96(96). doi: 10.3389/fneur.2020.00096 32158423 PMC7052123

[75] Battisti C Forte F Rubenni E Dotti MT Bartali A Gennari P. Two cases of hemichorea-hemiballism with nonketotic hyperglycemia: a new point of view. Neurol Sci (2009) 30(3):179–83. doi: 10.1007/s10072-009-0039-5 19305947

[76] Tan Y Xin X Xiao Q Chen S Cao L Tang H. Hemiballism-hemichorea induced by ketotic hyperglycemia: case report with PET study and review of the literature. Transl Neurodegener (2014) 3(14). doi: 10.1186/2047-9158-3-14 PMC4100749 25031834

[77] Postuma RB Lang AE. Hemiballism: revisiting a classic disorder. Lancet Neurol (2003) 2(11):661–8. doi: 10.1016/s1474-4422(03)00554-4 14572734

[78] Chen C Zheng H Yang L Hu Z. Chorea-ballism associated with ketotic hyperglycemia. Neurol Sci (2014) 35(12):1851–5. doi: 10.1007/s10072-014-1968-1 25262066

[79] Satish PV Pujitha K Agrawal N Mathew T Vidyasagar S. Hemi-chorea in a patient with ketotic hyperglycemia: An unusual presentation. J Clin Diagn Res (2017) 11(5):OD24–OD5. doi: 10.7860/JCDR/2017/27266.9939 PMC5483740 28658838

[80] Hefter H Mayer P Benecke R. Persistent chorea after recurrent hypoglycemia. a case report. Eur Neurol (1993) 33(3):244–7. doi: 10.1159/000116946 8467847

[81] Dong M E JY Zhang L Teng W Tian L. Non-ketotic hyperglycemia chorea-ballismus and intracerebral hemorrhage: A case report and literature review. Front Neurosci (2021) 15:690761(690761). doi: 10.3389/fnins.2021.690761 34248493 PMC8260933

[82] Kandiah N Tan K Lim CC Venketasubramanian N. Hyperglycemic choreoathetosis: Role of the putamen in pathogenesis. Mov Disord (2009) 24(6):915–9. doi: 10.1002/mds.22277 19243026

[83] Wintermark M Fischbein NJ Mukherjee P. Unilateral putaminal CT. MR, and diffusion abnormalities secondary to nonketotic hyperglycemia in the setting of acute neurologic symptoms mimicking stroke. AJNR Am J Neuroradiol (2004) 25(6):975–6. PMC7975680 15205134

[84] Curro CT Nicocia G Ziccone V Ciacciarelli A Russo G Toscano A. Pimozide and pancreatic cancer in diabetic chorea: a case report. Int J Neurosci (2022) 132(12):1217–20. doi: 10.1080/00207454.2021.1879063 33491547

[85] Matsis PP Fisher RA Tasman-Jones C. Acute lithium toxicity–chorea, hypercalcemia and hyperamylasemia. Aust N Z J Med (1989) 19(6):718–20. doi: 10.1111/j.1445-5994.1989.tb00344.x 2483612

[86] Le Hir A Hak JF Gragueb-Chatti I Bobot M. Hypocalcemia-induced seizure with fahr's syndrome. J Nephrol (2022) 35(3):1047–8. doi: 10.1007/s40620-022-01260-w 35133618

[87] Jin D Yoon WT Suh BC Moon HS Chung PW Kim YB. Exacerbation of idiopathic paroxysmal kinesigenic dyskinesia in remission state caused by secondary hypoparathyroidism with hypocalcemia after thyroidectomy: evidence for ion channelopathy. Brain Dev (2012) 34(10):840–3. doi: 10.1016/j.braindev.2012.01.014 22361453

[88] Howdle PD Bone I Losowsky MS. Hypocalcaemic chorea secondary to malabsorption. Postgrad Med J (1979) 55(646):560–3. doi: 10.1136/pgmj.55.646.560 PMC2428083 514936

[89] Topakian R Stieglbauer K Rotaru J Haring HP Aichner FT Pichler R. Hypocalcemic choreoathetosis and tetany after bisphosphonate treatment. Mov Disord (2006) 21(11):2026–7. doi: 10.1002/mds.21094 16972252

[90] Warren JD Kimber TE Thompson PD. Hypocalcaemic chorea secondary to malabsorption. Aust N Z J Med (1998) 28(3):343. doi: 10.1111/j.1445-5994.1998.tb01959.x 9673747

[91] Martinez-Ramirez D Walker RH Rodriguez-Violante M Gatto EM. Review of hereditary and acquired rare choreas. Tremor Other Hyperkinet Mov (N Y) (2020) 10(24). doi: 10.5334/tohm.548 PMC7413136 32832197

[92] Randall RE Jr. Rossmeisl EC Bleifer KH. Magnesium depletion in man. Ann Intern Med (1959) 50(2):257–87. doi: 10.7326/0003-4819-50-2-257 13627700

[93] Sparacio RR Anziska B Schutta HS. Hypernatremia and chorea. a report of two cases. Neurology (1976) 26(1):46–50. doi: 10.1212/wnl.26.1.46 942769

[94] Ezpeleta D de Andres C Gimenez-Roldan S. Abnormal movements in a case of extrapontine myelinolysis. review of the literature. Rev Neurol (1998) 26(150):215–20. doi: 10.33588/rn.26150.981060 9563090

[95] Mann TP. Transient choreo-athetosis following hypernatraemia. Dev Med Child Neurol (1969) 11(5):637–40. doi: 10.1111/j.1469-8749.1969.tb01495.x 5349655

[96] Alarcon F Maldonado JC Rivera JW. Movement disorders identified in patients with intracranial tuberculomas. Neurologia (2011) 26(6):343–50. doi: 10.1016/j.nrl.2010.12.011 21345541

[97] Piccolo I Defanti CA Soliveri P Volonte MA Cislaghi G Girotti F. Cause and course in a series of patients with sporadic chorea. J Neurol (2003) 250(4):429–35. doi: 10.1007/s00415-003-1010-7 12700907

[98] Ravindran T Paneerselvam R Yabesh TA. Osmotic demyelination syndrome presenting with chorea. J Assoc Physicians India (2016) 64(4):89–90. 27734653

[99] Tang WY Gill DS Chuan PS. Chorea, a manifestation of hyponatraemia? Singapore Med J (1981) 22(2):92–3. 7268455

[100] Kyle K Bordelon Y Venna N Linnoila J. Autoimmune and paraneoplastic chorea: A review of the literature. Front Neurol (2022) 13:829076. doi: 10.3389/fneur.2022.829076 35370928 PMC8972589

[101] Panzer J Dalmau J. Movement disorders in paraneoplastic and autoimmune disease. Curr Opin Neurol (2011) 24(4):346–53. doi: 10.1097/WCO.0b013e328347b307 PMC3705177 21577108

[102] Ghosh R Dubey S Roy D Ray A Pandit A Ray BK. Choreo-ballistic movements heralding COVID-19 induced diabetic ketoacidosis. Diabetes Metab Syndr (2021) 15(3):913–7. doi: 10.1016/j.dsx.2021.04.010 PMC8062421 33915346

[103] Klawans HL Jr. Shenker DM. Observations on the dopaminergic nature of hyperthyroid chorea. J Neural Transm (1972) 33(1):73–81. doi: 10.1007/BF01244729 4264577

[104] Baba M Terada A Hishida R Matsunaga M Kawabe Y Takebe K. Persistent hemichorea associated with thyrotoxicosis. Intern Med (1992) 31(9):1144–6. doi: 10.2169/internalmedicine.31.1144 1421727

[105] Hamed SA. Neurologic conditions and disorders of uremic syndrome of chronic kidney disease: presentations, causes, and treatment strategies. Expert Rev Clin Pharmacol (2019) 12(1):61–90. doi: 10.1080/17512433.2019.1555468 30501441

[106] Schrank S Barrington N Stutzmann GE. Calcium-handling defects and neurodegenerative disease. Cold Spring Harb Perspect Biol (2020) 12(7):a035212. doi: 10.1101/cshperspect.a035212 31427373 PMC7328457

[107] Barry J Peng A Levine MS Cepeda C. Calcium imaging: A versatile tool to examine huntington's disease mechanisms and progression. Front Neurosci (2022) 16:1040113. doi: 10.3389/fnins.2022.1040113 36408400 PMC9669372

[108] Tison FX Ferrer X Julien J. Delayed onset movement disorders as a complication of central pontine myelinolysis. Mov Disord (1991) 6(2):171–3. doi: 10.1002/mds.870060215 2057010

